# Protective effects of dietary grape against atopic dermatitis-like skin lesions in NC/NgaTndCrlj mice

**DOI:** 10.3389/fimmu.2022.1051472

**Published:** 2023-01-19

**Authors:** Chandra K. Singh, Charlotte A. Mintie, Mary A. Ndiaye, Gagan Chhabra, Sushmita Roy, Ruth Sullivan, B. Jack Longley, Stefan M. Schieke, Nihal Ahmad

**Affiliations:** ^1^ Department of Dermatology, University of Wisconsin, Madison, WI, United States; ^2^ Department of Comparative Biosciences, University of Wisconsin, Madison, WI, United States; ^3^ William S. Middleton Veterans Affairs (VA) Medical Center, Madison, WI, United States

**Keywords:** grapes (Vitus vinifera L.), atopic dermatitis (AD), skin lesions, acute phase response proteins, proteomics

## Abstract

Atopic dermatitis (AD) is a chronic inflammatory skin disease with significant health/economic burdens. Existing therapies are not fully effective, necessitating development of new approaches for AD management. Here, we report that dietary grape powder (GP) mitigates AD-like symptoms in 2,4-dinitrofluorobenzene (DNFB)-induced AD in NC/NgaTndCrlj mice. Using prevention and intervention protocols, we tested the efficacy of 3% and 5% GP-fortified diet in a 13-weeks study. We found that GP feeding markedly inhibited development and progression of AD-like skin lesions, and caused reduction in i) epidermal thickness, mast cell infiltration, ulceration, excoriation and acanthosis in dorsal skin, ii) spleen weight, extramedullary hematopoiesis and lymph nodes sizes, and iii) ear weight and IgE levels. We also found significant modulations in 15 AD-associated serum cytokines/chemokines. Next, using quantitative global proteomics, we identified 714 proteins. Of these, 68 (normal control) and 21 (5% GP-prevention) were significantly modulated (≥2-fold) vs AD control (DNFB-treated) group, with many GP-modulated proteins reverting to normal levels. Ingenuity pathway analysis of GP-modulated proteins followed by validation using ProteinSimple identified changes in acute phase response signaling (FGA, FGB, FGG, HP, HPX, LRG1). Overall, GP supplementation inhibited DNFB-induced AD in NC/NgaTndCrlj mice in both prevention and intervention trials, and should be explored further.

## Introduction

Atopic dermatitis (AD) is a chronic inflammatory and relapsing skin disease affecting ~9.6 million children and ~16.5 million adults in the United States (1, 2). AD can significantly impair the quality of life due to vicious cycles of intense itching/scratching, insomnia, depression and anxiety and has a considerable economic burden on our society, with more than $5 billion in annual management costs (3). AD is a complex disorder caused by the interplay between genetic and environmental factors (4) and characterized by abnormal immune function and skin barrier dysfunction (5). AD can manifest in an “atopic triad” along with rhinitis and asthma (1). The aggravation of disease on defective skin barrier can be epidemiologically linked to air pollutants such as tobacco smoke, formaldehyde and particulate matter (reviewed in (6)). The treatments for AD have been mostly reactive. Immune-suppressive topical or systemic steroids to control inflammation quickly result in AD improvement and have been in use for decades; however, their long-term usage results in an extensive range of adverse effects, such as skin atrophy, telangiectasia, hypertrichosis, and topical steroid addiction (7, 8). Newer therapies based on novel pathways or immune targets involved in AD pathogenesis are being identified and/or in use (reviewed in (9)). Additionally, strategies based on diet and/or lifestyle changes may be an alternative or supplemental strategy to help in the successful management of AD.

Naturally occurring botanicals have been shown to possess strong potential in the management of skin diseases (10–12). Several antioxidants found in grapes have been shown individually to be beneficial against dermatological conditions, including AD (12). Resveratrol, one of the most studied active agents of grape and grape-derived products, has been shown to exert beneficial effects on AD by modulating inflammation *via* acting on epithelium-derived cytokines and reducing dermal destruction in multiple mouse models of AD (13–15). Similarly, quercetin, another active phytochemical in red grapes has demonstrated the ability to inhibit AD in mice models (16–18). In this study, we determined the effect of dietary grape powder (GP) on AD development and progression in NC/NgaTndCrlj mice. These mice are known to develop skin lesions resembling human AD when housed in non-ventilated conditions as well as when purposely induced by irritants (19–21). The key characteristics of this model are the increased number and degranulation of mast cells and recruitment of inflammatory cells, as well as exacerbation of dermatitis related to the hyperproduction of immunoglobulin E (IgE). The rationale of this study is grounded in the fact that grape antioxidants in their natural combination (in whole grape) may have better efficacy against AD, owing to synergistic interactions among the many different components present in grapes.

## Materials and methods

### Dietary grape feed

Freeze-dried grape powder (GP) prepared from fresh red, green and black grapes by California Table Grape Commission (CTGC) was obtained and formulated in AIN-76A base diet by Envigo. Diets were formulated by Envigo as 0%, 3% and 5% GP (w/v), sugar matched to the natural content of 5% GP, and air-dried before shipment. The selection of grape powder doses was as per CTGC recommendation for animal feeding studies and has been used in several other studies (22).

### NC/NgaTndCrlj mice and animal experimentation

**Figure 1:**
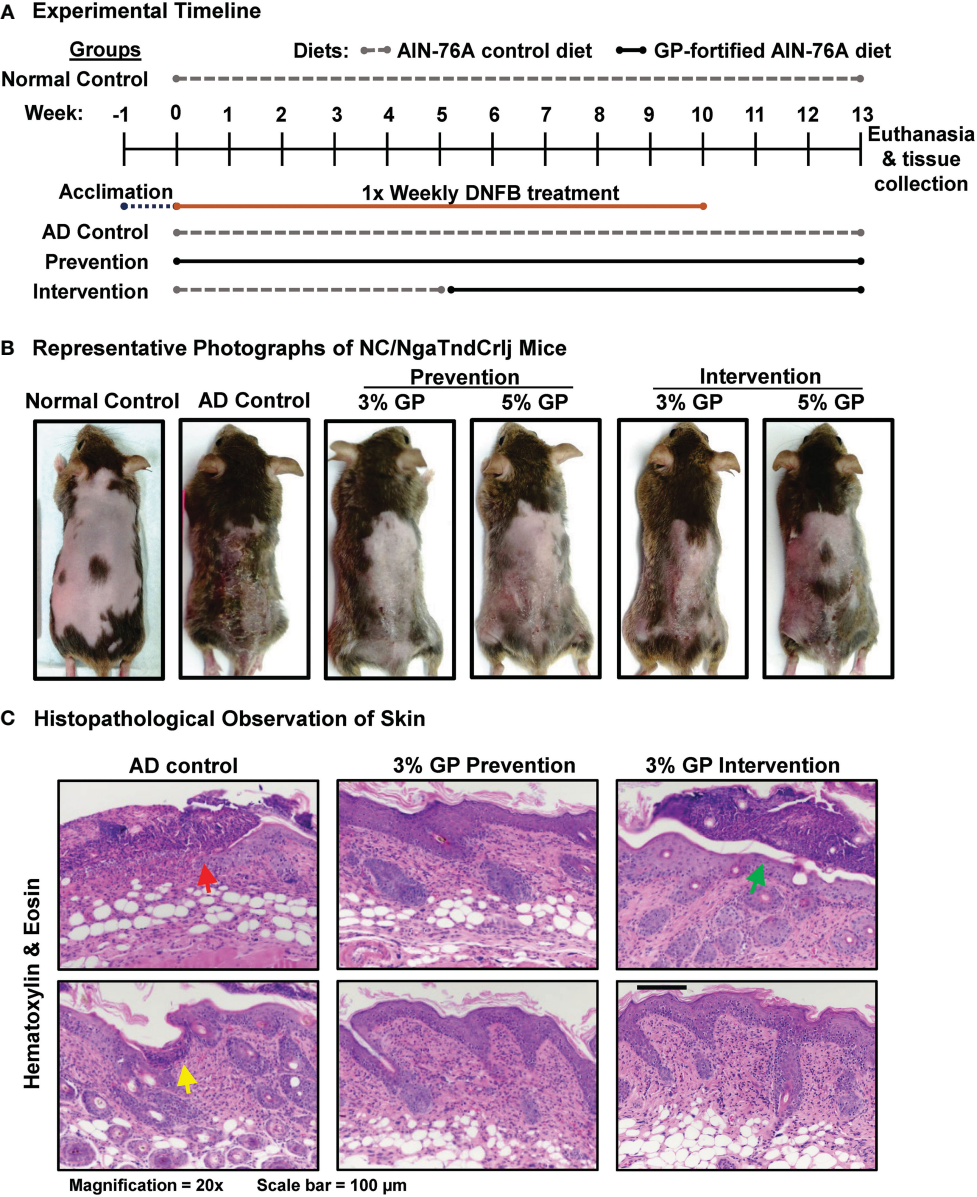
Effect of grape powder supplementation against DNFB-induced atopic dermatitis in NC/NgaTndCrlj mice. **(A)** Timeline and experimental design of DNFB-induced AD in NC/NgaTndCrlj mice, and prevention and intervention trials with 3% and 5% GP. **(B)** Representative images of mice from each experimental group immediately before euthanasia, demonstrating the severity of AD-like skin lesions across all groups. **(C)** AD histopathological analysis in skin sections was performed as described in ‘Materials and Methods’ section. At least five images were taken at 20x magnification across five different skin sections of each mouse. Representative images are shown.

Animal experiments were approved by the University of Wisconsin (UW) Institutional Animal Care and Use Committee. NC/NgaTndCrlj mice (female, n=8 per group) were purchased from Charles River Laboratories at 4 weeks of age and allowed to acclimate for one week before study initiation. Animals had access to water and feed ad libitum. As described elsewhere (23), AD was induced by weekly topical application of 150 µL of 0.15% DNFB in acetone/olive oil (Sigma; 3:1) to the hair-removed back skin of mice. A subset of mice was left in ventilated housing with no DNFB application, termed normal control. Mice within the prevention group received 3% or 5% GP-fortified AIN-76A diet from weeks 1-13 (3% GP-P and 5% GP-P), whereas mice within the intervention group did not receive GP-fortified feed until after the onset of AD-like lesions, at week 5 (3% GP-I and 5% GP-I) (**Figure 1A**). AD control mice received the same diet without GP. Throughout the study, mice were monitored for general health, body weight, food consumption as well as disease severity. After week 13, mice were euthanized and lesional dorsal skin and corresponding skin from normal control were collected for further analysis. Spleens and lymph nodes were also collected and imaged. The cross-sectional area of lymph nodes was measured using the formula (L/2*W/2*π). Spleens were weighed on an analytical balance. Ear tissues were collected using a 4 mm biopsy punch (three ear punches from each ear), weighed on an analytical balance and averaged per animal. Blood was collected by cardiac puncture immediately after euthanasia, and allowed to clot at room temperature, followed by centrifugation and collection of serum.

### Histologic evaluation

Harvested lesional dorsal skin, non-lesional ventral skin and spleens were formalin-fixed, paraffin-embedded, sectioned (at 5 µm), and mounted on slides by the Translational Research Initiatives in Pathology (TRIP) Lab, UW-Madison. Histopathological analyses were performed using hematoxylin/eosin (H&E) and toluidine blue (TB) dye to assess epidermal hyperplasia and degranulation of mast cells, as described earlier (24). Epidermal thickness was evaluated and images were acquired using EVOS FL Cell Imaging System (ThermoFisher Scientific). Mast cells were quantified from TB staining and averaged from five separate fields. Histological evaluation was performed by our collaborating pathologists Ruth Sullivan and B. Jack Longley. Stained tissue sections were imaged on EVOS FL Cell Imaging System.

### Immunoglobulin E (IgE) enzyme-linked immunosorbent assay (ELISA) and cytokine array analyses

Two biological replicates per group were made by pooling serum from four random mice and experiments were run in triplicate. Serum IgE levels were measured using mouse IgE ELISA (Abcam) per manufacturer’s instructions. Cytokine and chemokine levels in serum were analyzed using mouse Cytokine & Chemokine 36-Plex ProcartaPlex 1A panel (ThermoFisher Scientific) per manufacturer’s instructions. The assay was run using Luminex MagPix system (Luminex Corporation) and analyzed using ProcartaPlex Analyst software (ThermoFisher Scientific). Data is represented as Log2 (mean concentration of treatment (pg/mL)/mean concentration of control).

### Flow cytometry analysis

Lymph nodes collected from different treatment groups were stored in 10% formalin. Single cell suspension was prepared by crushing the tissue with a plunger on a 70 µ filter mesh. Live cells were counted and 1x10^6^ cells were taken from respective mice followed by blocking with 3% bovine serum albumin (BSA) and staining with IgE and IL4 antibodies (BD Pharmingen) for 30 min on ice. Cells were washed with 1x phosphate-buffered saline (PBS) containing 0.5% BSA, and immediately analyzed on Attune Flow Cytometer (ThermoFisher) in the UW Carbone Cancer Center (UWCCC) Flow Cytometry Laboratory. Data analysis were performed using Flowjo™10 software (BD Biosciences).

### Quantitative immuno-detection analyses using Jess ProteinSimple

Tissue lysates were prepared from dorsal skin tissues and protein quantification was performed using standard techniques as described earlier (24). Protein lysates from two mice were pooled in each group to make three groupings of protein for further analysis. The quantitative immunodetection by ProteinSimple was performed as described recently (25). Primary antibodies used in ProteinSimple analyses are detailed in **Supplementary Table S1**.

### Proteomics analysis

The quantitative proteomics was performed at the School of Pharmacy Analytical Instrumentation Facility, UW-Madison, as described earlier (26). Briefly, sample protein (20 µg) was digested with 1 µg sequencing grade trypsin and analyzed by nano-LC/MS/MS. The data were searched against the Swiss-Prot mouse proteome database using Sequest HT search engine in the Proteome Discoverer 1.4 software, and data were aligned using ChromAlign algorithm. Quantitation of peptides was performed on processed data using SIEVE 2.1 (ThermoFisher Scientific). The resultant proteomics data was deposited to the NIH public repository MassIVE (MSV000090686 [https://massive.ucsd.edu/ProteoSAFe/dataset.jsp?accession=MSV000090686]).

### RT-qPCR analysis

RT-qPCR analyses were done using standard techniques as described earlier (24). Briefly, RNA was isolated using RNeasy Mini Kit (Qiagen), and equal amounts were pooled from two mice to make three groupings of RNA for cDNA synthesis. RT-qPCR was performed using SYBR Premix Ex Taq II (TaKaRa) and appropriate primer sets (LRG1, TSLP, IL6 and ACTB) (**Supplementary Table S2**). The primer sequences were retrieved from PrimerBank (27). ΔΔCT comparative method using ACTB endogenous control was used to calculate relative mRNA levels.

### Statistical analysis

Statistical analyses for all the quantitative data were performed using GraphPad Prism 8 software (GraphPad Software, Inc.). All the analyses were compared with AD control. Unless specified elsewhere, comparisons between experimental groups were conducted using one-way analysis of variance (ANOVA) followed by Dunnett’s multiple comparisons method.

## Results

### Dietary grape powder (GP) inhibits DNFB-induced atopic dermatitis-like skin lesions in NC/NgaTndCrlj mice

To determine the effects of dietary grapes against AD in NC/NgaTndCrlj mice, we used two distinct protocols: prevention and intervention. The experimental timeline for DNFB-induced AD mouse model indicating when diets were provided, and length of study is detailed in **Figure 1A**. A graphical presentation of experimental strategies used in this study is provided in **Supplementary Figure S1**. In the prevention protocol, mice were provided with 3% or 5% GP-fortified diet at the beginning of study to determine if GP could reduce the onset and severity of the disease. In the intervention protocol, mice were given control diet until the onset of disease, then replaced with 3% or 5% GP-fortified diet to determine the therapeutic effects of dietary grape. The rationale for the time points chosen here is based on the fact that these mice develop AD in response to allergens from 6 to 8 weeks of age (19). Images of all NC/NgaTndCrlj mice (n=8 per group) at the end of study (13 weeks) are shown in **Supplementary Figure S2**. Representative photographs are shown in **Figure 1B**, with lesions most apparent in control group and visibly reduced in all treatment groups. We found that GP supplementation was well tolerated by mice, and no adverse events or body weight changes were noticed in response to GP supplementation (**Supplementary Figure S3**).

Following the course of treatments, skin demonstrated extensive crusting and excoriation on gross evaluation of AD control mice (**Figure 1B**), which corresponded to histologic findings of cutaneous ulceration and excoriation, extensive serocellular crust formation, hyperkeratosis and acanthosis, and dermatitis with both neutrophilic infiltrates in dermis and serocellular crusts (**Figure 1C**, red arrow). This was accompanied by lymphoid infiltrates in dermis and epidermis. Extensive neutrophilic inflammation was interpreted as an inflammatory response to the observed cutaneous ulceration and excoriation, and termed secondary effects. This was likely the result of mice scratching at regions of treated skin (predicted from small ulceration, suggestively from toenail (**Figure 1C**, yellow arrow). Typical lesions of acute AD such as spongiosis and parakeratotic hyperkeratosis were not observed, most likely due to late time-point of the evaluation (when spongiosis is typically resolved) and super-imposition of skin excoriations and inflammatory responses they incited. Evaluation of GP prevention animals demonstrated reduced presence of neutrophils, ulceration and excoriation, minimal crusts, and moderate hyperkeratosis and acanthosis (**Figure 1C**). Likewise, intervention groups appeared less affected by secondary effects as compared to control, but had minor serocellular crust formation (**Figure 1C**, green arrow). Overall, GP supplementation markedly inhibited the development of AD-like skin lesions in the prevention group and reduced the progression of lesions when provided as an intervention therapy. Specifically, GP was found to lessen AD severity as there were marked decreases of edema, erythema/hemorrhage, excoriation/erosion and dryness/scaling in GP-supplemented mice. GP supplementation also reduced severe AD-associated histopathological changes. Mice within normal control group showed no sign of AD development.

### GP supplementation decreases epidermal thickness and mast cell infiltration in NC/NgaTndCrlj mouse skin

**Figure 2:**
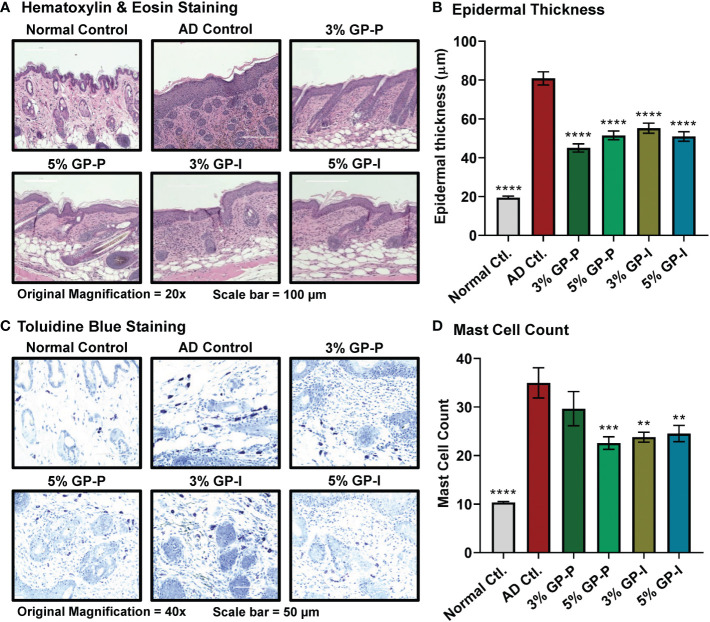
Effect of grape powder supplementation against atopic dermatitis-associated changes within skin of NC/NgaTndCrlj mice. **(A)** Tissue sections were stained with H&E and epidermal thickness was analyzed by measuring the distance from stratum basale to stratum corneum using EVOS FL Cell Imaging System. Representative images are shown. **(B)** ImageJ software (NIH) was used to average 25 measurements across 5 images at 20x magnification, then averaged per mouse. **(C)** Tissue sections were stained with toluidine blue for mast cell (violet metachromatic cytoplasmic granules) infiltration analysis. At least five images were taken at 40x magnification across five different skin sections of each mouse. Representative images are shown. **(D)** Mast cells were counted using ImageJ, then averaged per mouse. The quantitative data represents mean ± SEM of five animals per group. All the analyses were compared with AD control. A one-way ANOVA with Dunnett’s multiple comparisons test was performed (**p<0.01, ***p<0.001, ****p<0.0001).

To quantify the effects of GP consumption on epidermal hyperplasia, measurements of the epidermal layer from stratum basale to stratum corneum were obtained using the following criteria: 1) hair follicle appeared in correct orientation, 2) basal layer was present, 3) no ulceration/serocellular crust was present. We found that DNFB increased epidermal thickness nearly 4-fold in AD control compared to normal control group (80.92 ± 3.23 and 19.55 ± 0.96 μm, respectively). Additionally, both doses of GP significantly reduced the effects of DNFB treatment on epidermal thickening in both prevention and intervention groups (**Figures 2A, B**). This suggests that GP may not only aid in reducing the primary effects of AD but could help prevent scratching that can lead to traumatizing secondary effects within skin.

Due to the observation that chronic AD lesions present increased perivascular populations of lymphocytes and mast cells (28), we evaluated mast cell populations using toluidine blue staining. For this analysis, total number of mast cells was counted regardless of granulation. The mast cell counts indicated that GP aided inflammatory relief, as seen through marked reduction in 3% GP prevention group and significant reduction in 5% GP prevention and 3% and 5% GP intervention groups (**Figures 2C, D**). Overall, our analysis found significant GP-mediated reduction in epidermal thickness and mast cell infiltration in both prevention and intervention studies, suggesting GP-mediated protection against skin barrier dysfunction and abnormal immune responses in AD skin.

### GP supplementation reduces extramedullary hematopoiesis in spleen and lymphadenopathy

**Figure 3:**
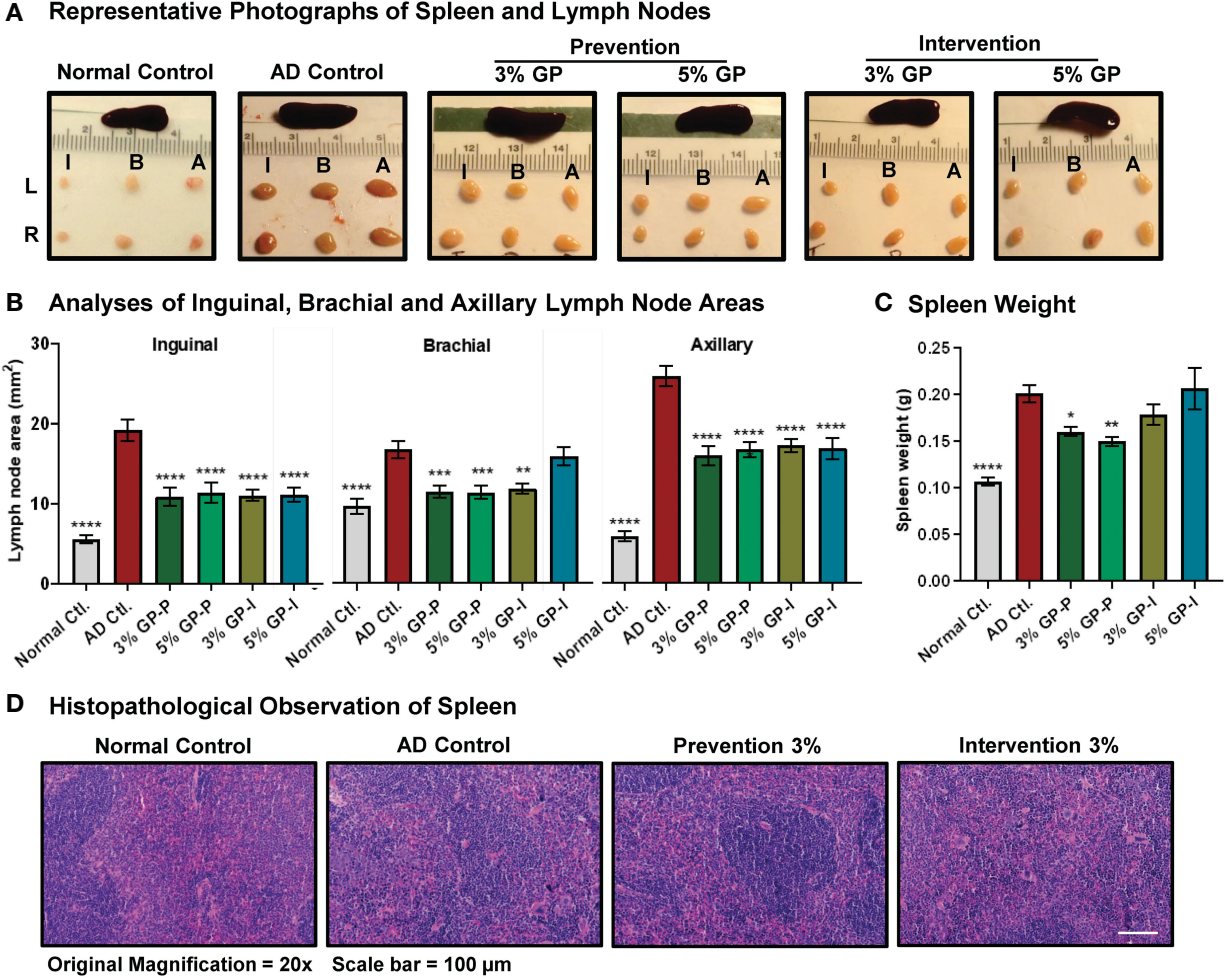
Effect of grape powder supplementation on extramedullary hematopoiesis in spleen and lymphadenopathy in atopic dermatitis. **(A)** After euthanasia, spleen and lymph nodes (left (L) and right (R) inguinal (I), brachial (B) and (A) axillary) were collected and imaged. **(B)** The cross-sectional area of all collected lymph nodes was measured using the formula (L/2*W/2*π), and plotted. **(C)** The weight of spleens was measured using an analytical scale. All the analyses were compared with AD control. A one-way ANOVA with Dunnett’s multiple comparisons test was performed and represented as mean ± SEM with statistical significance (*p<0.05, **p<0.01, ***p<0.001, ****p<0.0001). **(D)** Spleen histopathological analyses were performed by our collaborating pathologist Ruth Sullivan as described in ‘Result’ section. Representative images at 20× magnification are shown.

As AD is an inflammatory skin disease, spleen and lymph nodes were collected and analyzed. Splenomegaly (enlarged spleen) and lymphadenopathy (enlarged lymph nodes) were observed in mice with AD disease, which were mitigated in response to GP supplementation (**Figure 3A**). Our analysis found significant decreases in all three lymph nodes (inguinal, brachial and axillary) of GP-supplemented mice (except for brachial 5GP intervention group) (**Figure 3B**). Spleen weights were significantly increased in AD control mice compared to normal control counterparts (**Figure 3C**). This increase was partially mitigated in animals in the prevention protocol, but not in the intervention protocol (**Figure 3C**). Spleen in mice is both a hematopoietic and lymphoid organ and plays important roles both in hematopoietic response to physiologic demand for blood cells, as occurs during chronic inflammation and in lymphoid immune responses (29). Histologic examination of spleens of AD mice revealed typical tissue architecture of red and white pulp; splenic enlargement occurred with retention of normal micro-architecture and thus is most likely a physiologic response to demand for blood cells (**Figure 3D**). Although quantitation of cellular compartments of spleen was not possible based on histology, there was a tendency for red pulp hematopoietic zones to be noticeably expansive in mice with marked skin disease. Because we did not undertake the quantitative analysis of cellular composition of enlarged spleens in this study, we cannot define the relative contributions of expanded red vs white pulp to splenic enlargement.

### GP supplementation modulates immunoglobulin E (IgE) and AD-associated cytokines and chemokines

**Figure 4:**
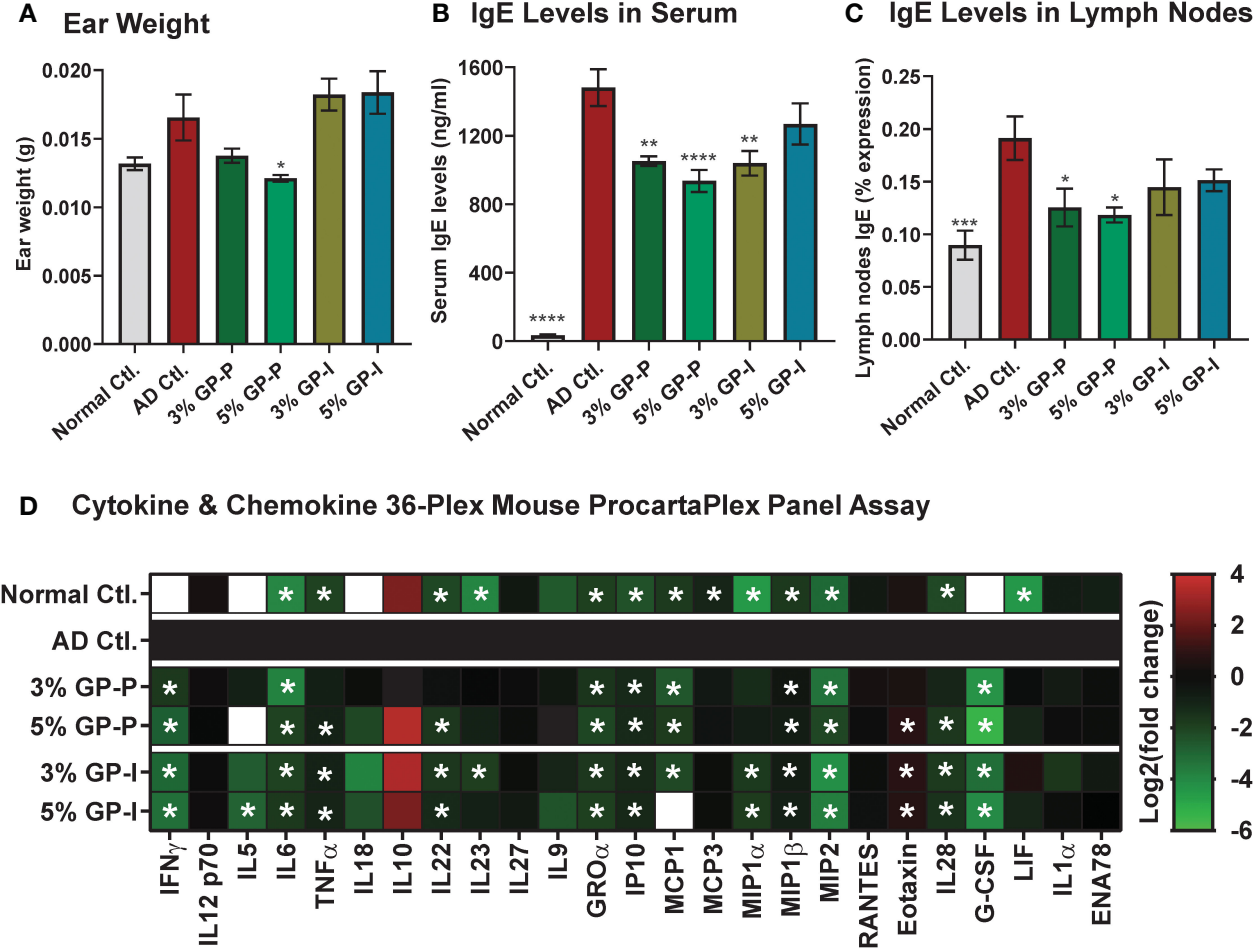
Effect of grape powder supplementation on the ear weight, lgE levels, and key cytokines and chemokines in atopic dermatitis. **(A)** At the time of tissue collection, three ear punches were obtained from each ear using a biopsy punch. Punches were weighed and averaged per animal. **(B)** Blood was collected by cardiac puncture after euthanasia, and serum was extracted. Serum levels of IgE were measured using ELISA. **(C)** Lymph nodes IgE was measured using Flow cytometry as described in ‘Materials and Methods’ section. **(D)** Serum cytokines and chemokines were measured using Cytokine & Chemokine 36-Plex Mouse ProcartaPlex Panel. The assays with serum were performed using a pool of 2 mice per sample in biological quadruplicate. Ear weight and IgE data are represented as mean ± SEM. ProcartaPlex Panel data are presented as Log2 (mean concentration of experimental groups (pg/mL)/mean concentration of AD control). A one-way ANOVA with Dunnett’s multiple comparisons test was performed and represented as mean ± SEM with statistical significance compared to AD control (*p<0.05, **p<0.01, ***p<0.001, ****p<0.0001).

As inflamed ears and IgE hyperproduction are key features of AD pathogenesis, we analyzed ear punch weight along with serum IgE levels *via* ELISA. A marked decrease was noticed in the ear weight of 3% and 5% GP prevention groups, but not in the intervention groups (**Figure 4A**). Regarding IgE, a drastic increase was noticed in AD control group as compared to normal control mice, which was significantly attenuated by GP at both doses in prevention study and within 3% GP intervention group (**Figure 4B**). IgE expression levels were also analyzed in lymph nodes as IgE is produced by plasma cells located in lymph nodes and lymphadenopathy was seen in our AD experimental model. Similar to serum IgE, lymph nodes IgE was significantly increased in AD control group (compared to normal control) with a significant decrease in response to 3% and 5% GP supplementation (compared to AD control group) in prevention groups and a decreasing trend in intervention groups, further corroborating our findings (**Figure 4C**).

In AD inflammatory environment, cytokines are generally upregulated (30). We analyzed levels of 36 immune targets in serum using the Cytokine & Chemokine 36-Plex Mouse ProcartaPlex Panel assay. The rationale to use this cytokine panel was based on its inclusion of diverse cytokines, including those related to Th1/Th2 and Th9/Th17/Th22/Treg, as well as inflammatory cytokines and chemokines, which allowed us to assay a broad range of targets at the same time with minimal sample volume. Out of 36, 25 cytokines were detected and quantified in most experimental groups (**Supplementary Figure S4**), however, only 15 cytokines showed significant modulation in serum samples derived from GP-supplemented mice (**Figure 4D**). The data showed significant decrease of serum cytokines related to Th1/Th2 (IFNγ, IL5, IL6, TNFα), and Th9/Th17/Th22/Treg (IL22, IL23) targets in GP-supplemented mice. A decrease in response to GP supplementation was also noticed in inflammatory cytokines (IL28, G-CSF) and chemokines (GROα, IP10, MCP1, MIP1α, MIP1β, MIP2) with an exception of increased chemokine Eotaxin. These responses were quite consistent at both doses of GP in prevention and intervention groups, and appeared to trend towards normalization level i.e. comparable to normal control group (**Figure 4D** and **Supplementary Figure S4**). A literature analysis outlining the relevance of these cytokines/chemokines in AD pathogenesis and/or inflammation is presented in **Table 1**. We also analyzed IL-4 in lymph nodes, which is known to promote Th2 lymphocyte development and generally elevated levels are seen in AD patients and mouse models (45). We found a significant increase in lymph nodes IL4 in AD and significant inhibition in response to GP supplementation in both prevention and intervention groups (**Supplementary Figure S5**). The cytokine & chemokine panel also had IL-4 as a part of 36-Plex, however it was only detected in the serum samples of AD control mice, and not in any of the treatment groups or normal control (data not shown). Next, we analyzed pro-Th2 cytokine thymic stromal lymphopoietin (TSLP), which is known to impair epidermal barrier integrity and its increased expression is seen in lesional skin of AD patients (46). Using RT-qPCR analysis, we found a significant increase in TSLP in AD skin tissues and decrease in response to GP, further suggesting modulation of proinflammatory cytokines in response to GP supplementation (**Supplementary Figure S6**).

**Table 1:** A literature analysis of 14 cytokines/chemokines identified using Cytokine & Chemokine 36-Plex Mouse ProcartaPlex Panel assay for their roles in atopic dermatitis (AD) and/or inflammation.

Symbols	Cytokines /Chemokines	Roles in AD and/or inflammation
Cytokines-related Th1/Th2 targets		
IFNγ	Interferon- gamma	Contributes to AD severity by impairing the epidermal barrier by affecting fatty acid composition of ceramide (a component of stratum corneum) (31).
IL5	Interleukin 5	Critical to eosinophils which are common in inflammatory infiltrate of AD (32).
IL6	Interleukin 6	Acts in inflammation. Anti-inflammatory treatments, e.g. quercetin reduces the expression of IL6 and helps control AD symptoms (17).
TNFα	Tumor necrosis factor-alpha	TNFα decreases long-chain free fatty acids and ester-linked ω-hydroxy (EO) ceramides, consequently affecting epidermal barrier function in AD (33).
Cytokines-related Th9/Th17/Th22/Treg targets		
IL22	Interleukin 22	IL22 inhibits the expression of key barrier molecules, therefore, is involved in barrier dysfunction in AD. IL22 inhibition shows profound effects on multiple inflammatory pathways in AD (34).
IL23	Interleukin 23	IL23 works with IL17 and is an important target therapy in inflammatory diseases, including AD (35).
Chemokines:		
GROα (CXCL1)	Growth- regulated oncogene- alpha	Contribute to AD pathogenesis, acts as a growth factor and is significant in inflammation and chemoattractant of neutrophils (36).
IP10 (CXCL10)	Interferon- inducible protein 10	IFNγ, TNFα and IL18 are potent stimulators of IP10. Increased IP10 has been seen in AD lesions (37).
MCP1 (CCL2)	Monocyte chemo-attractant protein- 1	Regulate the immune system and inflammatory processes. Beta-carotene has been seen to suppress MCP1 in skin tissues and improves barrier function in AD (38).
MIP1α (CCL3)	Monocyte chemoattractant protein 1α	Elevated in AD patients and patients with other inflammatory skin diseases, and levels decrease with therapy (39).
MIP1β (CCL4)	Monocyte chemoattractant protein 1β	Upregulation is generally seen at the source of inflammation to activate neutrophils, including in AD (40).
Eotaxin (CCL11)	Eotaxin	Play an important role in inflammation and AD pathogenesis (41). Its increase in response to GP, but no change compared to normal control needs to be further explored in AD.
MIP2 (CXCL2)	Macrophage Inflammatory Protein 2	Play an important role in immunoregulatory and inflammatory processes. Higher expression of MIP-2 has been found in the dermis of AD mice (42).
Inflammatory cytokines:		
IL28	Interleukin 28	Participate in the adaptive immune responses. Its role has not been defined in AD.
G-CSF (CSF3)	Granulocyte colony-stimulating factor	G-CSF, which is overexpressed in lesional AD regulates the innate immune system and influences T-cell function and dendritic cell activation (43, 44).

All the cytokines/chemokines presented in this table were decreased in response to grape powder supplementation in *NC/NgaTndCrlj* mice, with an exception of increased chemokine Eotaxin.

### Quantitative proteomics followed by Ingenuity Pathway Analysis (IPA) identified key AD-and/or dermatological condition-associated signaling in response to GP supplementation.

**Figure 5:**
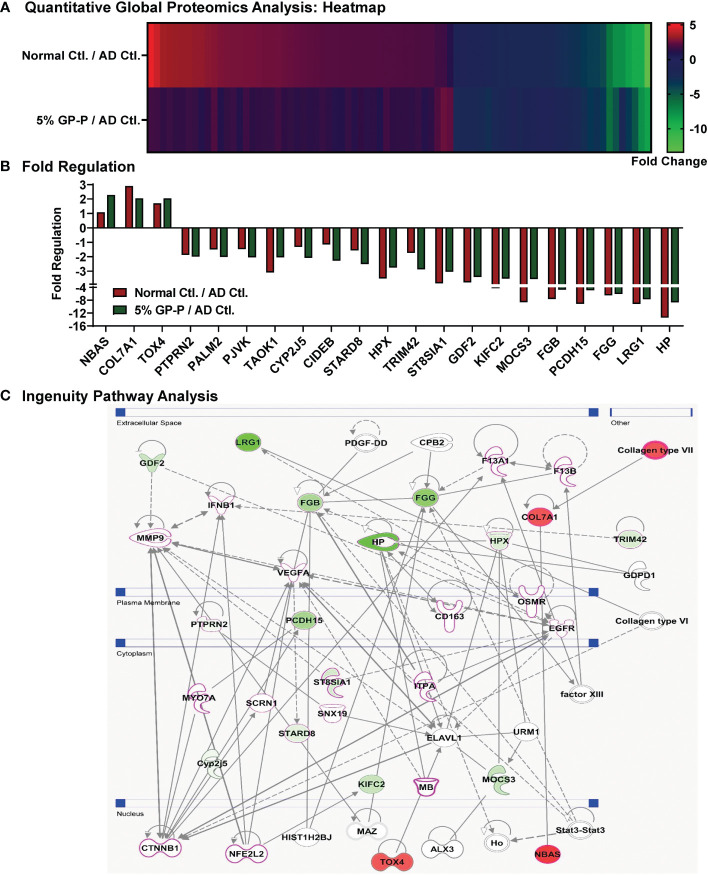
Quantitative global proteomics analyses of biological responses of grape powder supplementation against atopic dermatitis. Proteomics analyses from protein lysates (n=6 each) derived from normal control, AD control and 5% GP prevention were performed as described in ‘Materials and Methods’ and ‘**Figure S7**’ sections. **(A)** Heatmap of 79 proteins showing ≥2-fold change with statistical significance in 5% GP or normal control compared to AD control was plotted. **(B)** Compared to AD control, 21 of these proteins significantly modulated ≥2-fold in 5% GP group were plotted along with normal control for comparative analysis. **(C)** The 21 proteins identified in response to GP were subjected to network pathway analyses using IPA. The protein-protein interactions are indicated by arrows. The solid lines denote a robust correlation with partner genes, and dashed lines indicate statistically significant but less frequent correlations. The pink outline indicates that their role in AD/other dermatological conditions is known. Green fill indicates downregulated proteins and red fill indicates upregulated ones.

To uncover molecular mechanism(s) of biological responses of GP, dorsal skin samples from normal control, AD control and 5% GP prevention groups were analyzed using gel-free quantitative global proteomics analysis. An overview of proteomics experimental design and analyses are presented in **Supplementary Figure S7**. Overall, 6,987 unique peptides were identified with p-values <0.05, and 713 proteins were identified with ≥2 unique peptides and quantified (**Supplementary Table S3**). Compared to AD control, 68 of these proteins were significantly modulated ≥2-fold in normal control and 21 proteins in 5% GP group. A heat map showing significantly modulated proteins in normal control and 5% GP compared to AD control is represented in **Figure 5A**. Interestingly, several proteins identified in response to GP supplementation appear to trend towards a normalized level i.e. comparable to the normal control group (**Table 2** and **Figure 5B**). A literature analysis of these 21 proteins modulated by 5% GP outlining the relevance in AD pathogenesis and/or other dermatological conditions is presented in **Table 3**. Most of these proteins were not related to any known role in AD, however, their role in other skin conditions suggests that these proteins may have some role in AD and thus should be investigated further. These 21 proteins were further analyzed using IPA to identify novel potential target(s)/pathways that may be important in AD development and progression. Generating a network involving these proteins, as well as other associated proteins suggests modulation of major AD- and/or certain dermatological condition-associated signaling molecules viz. CTNNB1, NFE2L2, VEGFA, EGFR and MMP9 (indicated with pink outline) (**Figure 5C**).

**Table 2:** Grape powder (GP) modulated (≥2-fold) proteins identified using quantitative global proteomics analyses.

Protein ID	Protein Name	Location	Type(s)	Symbol	5% GP/AD control	Normal control/AD control
E9Q411	Neuroblastoma amplified sequence	Nucleus	other	NBAS	2.28	1.08
Q63870	Collagen type VII alpha 1 chain	Extracellular Space	other	COL7A1	2.05	2.89
Q8BU11	TOX high mobility group box family member 4	Nucleus	transcription regulator	TOX4	2.04	1.7
P80560	Protein tyrosine phosphatase, receptor type N2	Plasma Membrane	phosphatase	PTPRN2	-1.97	-1.87
Q8BR92	Paralemmin 2	Plasma Membrane	other	PALM2	-2.01	-1.49
Q0ZLH2	Pejvakin	Other	other	PJVK	-2.04	-1.48
Q5F2E8	TAO kinase 1	Cytoplasm	kinase	TAOK1	-2.04	-3.09
O54749	Cytochrome P450, family 2, subfamily j, polypeptide 5	Cytoplasm	enzyme	CYP2J5	-2.07	-1.32
O70303	Cell death-inducing DFFA-like effector b	Cytoplasm	other	CIDEB	-2.27	-1.15
Q8K031	StAR related lipid transfer domain containing 8	Cytoplasm	other	STARD8	-2.51	-1.56
Q91X72	Hemopexin	Extracellular Space	transporter	HPX	-2.75	-3.5
Q9D2H5	Tripartite motif containing 42	Other	other	TRIM42	-2.87	-1.74
Q64687	ST8 alpha-N-acetyl-neuraminide alpha-2,8-sialyltransferase 1	Cytoplasm	enzyme	ST8SIA1	-3.04	-3.84
Q9WV56	Growth differentiation factor 2	Extracellular Space	growth factor	GDF2	-3.4	-3.78
O08672	Kinesin family member C2	Cytoplasm	other	KIFC2	-3.52	-4.27
A2BDX3	Molybdenum cofactor synthesis 3	Cytoplasm	enzyme	MOCS3	-3.55	-8.58
Q8K0E8	Fibrinogen beta chain	Extracellular Space	other	FGB	-4.71	-7.61
Q99PJ1-13	Protocadherin related 15	Plasma Membrane	other	PCDH15	-4.9	-9.12
Q8VCM7	Fibrinogen gamma chain	Extracellular Space	other	FGG	-6.04	-6.49
Q91XL1	Leucine rich alpha-2-glycoprotein 1	Extracellular Space	other	LRG1	-7.67	-9.14
Q61646	Haptoglobin	Extracellular Space	peptidase	HP	-8.64	-13.4

**Table 3:** A literature analysis of proteins significantly affected by 5% grape powder supplementation in *NC/NgaTndCrlj* mice.

Protein	Fold change	Role in AD/other dermatological conditions
NBAS	2.28	Mutation in NBAS gene has been linked to multisystem disorders including reduced skin turgor and elasticity (47).
COL7A1	2.05	COL7A1 is essential for the production of type VII collagen precursor called pro-a1(VII) chain. Its inactivation in mice results in severe blistering phenotypes (48).
TOX4	2.04	TOX expression has been analyzed in cutaneous T-cell lymphomas (CTCL) and noticed that its expression is not tumor-specific or restricted to CD4+ CD8- phenotype (49).
PTPRN2	-1.97	A mutation in PTPRN2 has been linked to CTCL (50).
PALM2	-2.01	PALM2 is a protein anchored to the plasma membrane. Monoclonal antibody PALM2 has been demonstrated as a marker to discriminate between melanomas and nevocellular nevi (51).
PJVK	-2.04	PJVK, also known as DFNB59, is a member of gasdermin (GSDM) family generally expressed in skin and various immune cells, and mediates homeostasis and inflammation in response to caspases and proteases activation (52).
TAOK1	-2.04	TAOK1 is a serine/threonine-protein kinase generally involved in DNA damage responses and cytoskeletal stability. In human vitiligo cell line PIG3V, TAOK1 is highly upregulated. It contains miR-211 binding sites which influence pigmentation (53).
CYP2J5	-2.07	Predominantly expressed in the kidneys, CYP2J5 family proteins aid in amino acid metabolism. So far, CYP2J5 does not have a known role in any skin conditions.
CIDEB	-2.27	CIDEB is an endoplasmic reticulum and lipid droplet-associated protein, and a member of the CIDE family proteins that are vital regulators of lipid metabolism in the adipose tissue and skin sebaceous glands (54).
STARD8	-2.51	STARD8 is a Rho-GTPase activating protein that localizes to focal adhesions. Its role in skin conditions is not known.
TRIM42	-2.87	TRIM42 is a member of TRIMP superfamily generally known to express in response to IFNs and is associated with innate immunity (55).
ST8SIA1	-3.4	A higher level of ST8SIA1 (aka GD3 synthase) has been found in melanoma. It has also been found that inflammatory cytokines (e.g. TNFα and IL-6) secreted from UVB-irradiated keratinocytes enhances GD3 synthase gene expression (56).
GDF2	-3.4	GDF2 also known as BMP9 has recently been found to promote cutaneous wound healing (57). However, its role in AD is not known.
KIFC2	-3.52	KIFC2 plays a role in microtubule-dependent retrograde transport. Its role in skin conditions is not known.
MOCS3	-3.55	MOCS3 is necessary for the activation of molybdopterin synthase and molybdozneyme. Its role in skin conditions is not known.
PCDH15	-4.9	PCDH15 is known to mediate cell adhesion. Its role in skin conditions is not known.
LRG1	-7.67	LRG1 is an acute-phase protein related to inflammation-associated diseases and is known to be involved in protein-protein interaction, signal transduction, and cell adhesion (58). It promotes wound repair and keratinocyte migration *via* HIF-1α stability regulation (59). However, it also promotes melanoma dissemination *via* EGFR/STAT3 signaling regulation (60).
FGA	-2.0	The modulation in FGA was identified by immunodetection. This protein was included in our analysis because its elevated serum level has been noticed in AD patients (61).
FGB	-4.71	FGB along with FGA have been found to be upregulated in a bioengineered humanized acne microenvironment model (62).
FGG	-6.04	Along with FGA and FGB, FGG polymerizes to form an insoluble fibrin matrix, and functions during the initial stages of wound repair to stabilize lesions and guide cell migration during re-epithelialization. FGG does not have a known role in AD.
HP	-8.64	HP binds free plasma hemoglobin and prevents the loss of iron. HP levels were found to be increased in patients with psoriasis (63).
HPX	-2.75	Hemopexin has a high affinity for heme and is involved in the main route of heme elimination through complex binding. Serum hemopexin levels have a negative correlation with methemoglobin and a positive correlation with haptoglobin (HP) (64). So far, hemopexin does not have a known role in AD.

These proteins were identified during quantitative global proteomics and/or quantitative immunodetection analyses.

### GP supplementation attenuates acute phase response (APR) signaling.

**Figure 6:**
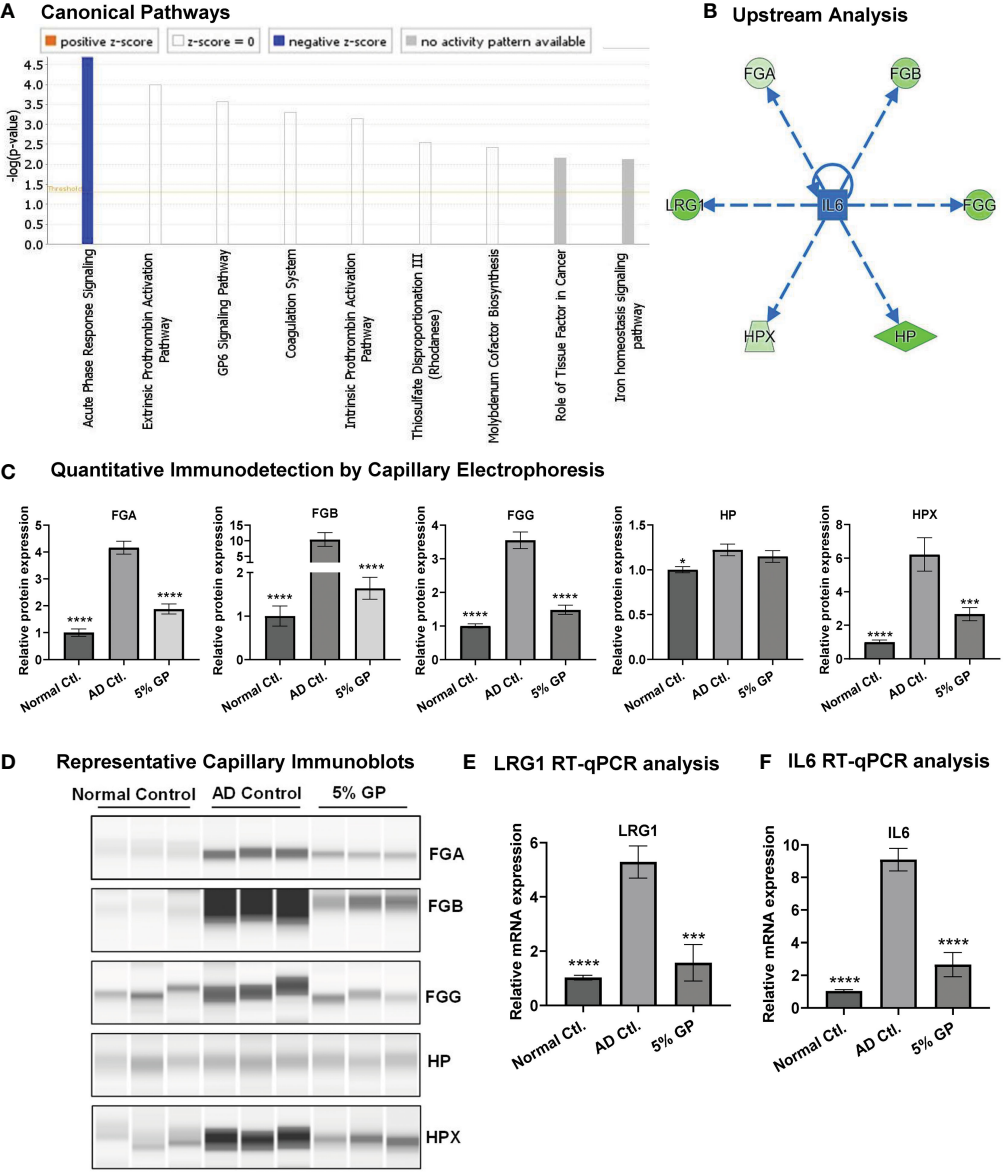
Effect of grape powder supplementation on acute phase response signaling in atopic dermatitis. **(A)** IPA was used to identify canonical pathways in responses to GP supplementation. Acute-phase response signaling was found with significant negative z-score. **(B)** IPA was also used to identify upstream regulator-related acute-phase proteins. **(C, D)** Validation of acute-phase proteins was performed using quantitative immunodetection by ProteinSimple Jess module as described in ‘Materials and Methods’. The protein quantitative data were normalized to the total capillary area of ‘Total Protein Assay’ of the same sample. **(E, F)** Validation of LRG1 and IL6 using RT-qPCR analysis. ACTB was used as an endogenous control. All the quantitative data were analyzed using one-way ANOVA followed by Dunnett’s multiple comparison test. The data are presented as mean ± SEM of three biological pools (n = 6) and three technical replicates with statistical significance compared to AD control (*p<0.05, ***p<0.01, ****p<0.0001).

IPA analysis of the proteomics data indicated the top canonical pathway modulated within the dataset to be APR signaling, suggesting inhibition of APR signaling in response to grape supplementation (**Figure 6A**). The identification of APR is an important finding because inflammation in AD leads to the influx of cytokines by surrounding cells, which prompts liver to produce acute-phase proteins (APPs), a set of early responses by body to acute damage. Our proteomics analysis identified significant inhibition in key APPs (fibrinogen beta (FGB), fibrinogen gamma (FGG), haptoglobin (HP), hemopexin (HPX) and leucine-rich alpha-2-glycoprotein 1 (LRG1)) that are linked to APR pathway (**Figure 5B**). Fibrinogen is a glycoprotein complex, composed of two trimers, with each trimer made of polypeptide chains, FGA, FGB and FGG (65). FGA was not identified during proteomics analysis but was included during IPA and ProteinSimple analysis as its elevated serum level has been noticed in AD patients (61). IPA analysis of GP-modulated proteins predicted the inhibition of interleukin 6 (IL6), which is a key player in AD pathogenesis (66). Interestingly, the inhibition of IL6 was predicted to be linked to the inhibition of APPs (**Figure 6B**). We validated the expression of APPs (FGA, FGB, FGG, HP and HPX) using quantitative immuno-detection analyses by ProteinSimple. We found significantly increased levels of FGA, FGB, FGG, HP and HPX in AD control, which were significantly diminished in response to 5% GP supplementation except for HP which showed a decreasing trend (**Figures 6C, D**). LRG1 was validated using RT-qPCR analysis and found significant increase in AD control tissues and decrease in response to 5% GP supplementation (**Figure 6E**). LRG1, fibrinogens (FGA, FGB, FGG) and transport proteins HP and HPX are positive APPs, which show enhanced expression in response to systemic inflammation, thus, lowered levels of these proteins in response to GP supplementation suggested reduced inflammation. The IPA-predicted inhibition of IL6 in response to GP was confirmed in AD skin tissues using RT-qPCR analysis. We found ~9-fold increase in IL6 in AD and significant decrease in response to 5% GP (**Figure 6F**). These findings suggested the role of IL6 and APPs in GP-mediated protection against AD.

## Discussion

AD is a chronic and relapsing skin disease affecting millions of people worldwide. It is a multifaceted disease and a number of signaling molecules are known to be involved in the pathogenesis of this disease (1, 7). Recent studies suggest that natural agents in combination could be useful in dealing conditions with complex modes of disease progression (67). It appears that the consumption of polyphenolic antioxidants in their natural matrix in whole foods may have implications for reducing the risk for several disease conditions, as individuals can modify their dietary habits and lifestyle to include these foods (reviewed in (11)). Arguably, grape is one of the most valuable fruits in the world, as it contains a plethora of antioxidants and micronutrients (68). Catechin, epicatechin, peonidin, cyanidin, malvidin, kaempferol, isorhamnetin, taxifolin, quercetin and resveratrol have been identified as top 10 compounds of GP, which constitute >70% of grape polyphenols (22, 68). Several grape antioxidants have been studied individually or in dual combinations against certain inflammatory skin conditions, including AD. For example, individually resveratrol and quercetin have been found to be beneficial against AD-like skin lesions (13, 14, 16, 18). Our study provides evidence that whole grape, likely due to the natural conglomeration of several antioxidants and micronutrients, may be a useful approach for AD management. Our data demonstrate that dietary supplementation of grapes inhibits AD-like skin lesions in a mouse model relevant to human AD (20, 21). In humans, acute AD is observed as eczematous patches and histologically observed as epidermal intracellular spongiosis with hyper- and para-keratosis, whereas chronic lesions have prominent perivascular infiltrate of leukocytes, epidermal hyperplasia and acanthosis, and hypertrophied upper layer (7, 28). The NC/NgaTndCrlj mouse model used in this study rapidly develops erythematous, erosive lesions with edema and hemorrhage that show hyperparakeratosis, hyperplasia and spongiosis in disease progression (28). The mouse photographs taken immediately before euthanasia (**Supplementary Figure S2**) and histopathological observation of AD-affected skin area (**Figures 1**, **2**) demonstrate the comparison of AD-like skin lesions across all treatment groups and suggest that GP supplementation markedly inhibited the development of AD-like skin lesions in prevention group and reduced the progression of lesions when provided as an intervention.

AD lesions are also characterized by increased infiltration of inflammatory cells such as mast cells and activated T helper cells (Th1 and Th2) (1, 5). Therefore, treatments that inhibit these cells could be useful against AD. The changes in the epidermis, which play key roles in immune surveillance, occur during AD pathogenesis (69). In our study, histopathological analyses of dorsal skin tissues showed an approximately four-fold increase in epidermal thickness and mast cell infiltration in AD mice, which were reduced at both doses of GP as a preventative and interventive agent. In AD control mice, we noticed splenomegaly and lymphadenopathy which are known to be manifested by a variety of inflammatory etiologies (70). We observed enlarged inguinal, brachial and axillary lymph nodes in mice with AD disease, which were alleviated in GP-supplemented mice at both doses in prevention and intervention settings. We found that spleen weights were increased in AD mice which were normalized in GP prevention but not in the intervention group, suggesting that secondary effects of the advanced disease have yet to recover lowering the threshold of immune cell infiltrate. Our histologic analysis of spleen suggested that splenic enlargement was a physiologic response (based on normal tissue architecture) and that expansive hematopoietic activity is likely to have contributed based on our qualitative analysis of spleen tissue. Enlargement of spleen due to the physiologic expansion of hematopoietic compartment has been reported in mice with skin disease (29), presumably due to the increased demand for white blood cells to participate in inflammatory responses. Previous studies have identified lymphoid expansion in white pulp of spleens of mice in several models of AD and other skin diseases, however, did not examine possible contributions of red pulp expansion in their models (71–73).

Cytokines are known to play critical role in AD as they cause barrier defects and inflammation culminating into the pathophysiology of this disease (74). Th2 immune response increases lgE levels and cytokine levels (75), ultimately leading to the production of damaging reactive oxygen species. Our data showed increased serum and lymph nodes IgE levels in AD mice which were alleviated in GP-supplemented mice, especially in prevention group. As AD pathogenesis is now understood to be much more heterogeneous, we implemented the analyses of multiple cytokines and chemokines using 36-Plex Mouse ProcartaPlex Panel assay for cytokines. Our data showed increased levels of several cytokines and chemokines associated with AD pathogenesis which were trending towards normalization level in response to GP supplementation in both prevention and intervention groups. AD has been mostly regarded as a Th2 signaling disease, however, there is some evidence that suggests the importance of Th1 signaling in AD (76). Our data showed decreased levels of serum cytokines related to Th2 (IL4 and IL5) as well as Th1 targets (IFNγ and TNFα) in response to GP supplementation. Since IL-4 was detected in the serum samples of AD control mice and but not in any of the treatment groups, we analyzed its level in lymph nodes, and found increased IL4 in AD lymph nodes which was mitigated in response to GP supplementation. Our data support a protective role of GP, as IL4 and IL5 contribute to AD pathogenesis (32, 45), and IFNγ and TNFα impair epidermal barrier function in AD (31, 33). IL-6 is known to promote Th2 differentiation and at the same time inhibit Th1 polarization (77). In this study, IL6 appeared as an important target which was increased in AD serum and skin samples and decreased in response to GP, and also appeared as a connecting target in APR signaling. Our data is in accordance with a previous publication where an individual grape antioxidant quercetin was shown to reduce IL6 in AD (17). Cytokines IL22 and IL23 which are related to Th9/Th17/Th22/Treg targets and display profound effects on multiple inflammatory pathways in AD (34, 35) showed a similar response to GP supplementation. Inflammatory cytokine G-CSF which is overexpressed in both epidermal and dermal compartments of lesional AD skin (43, 44) was not detected in normal control, but hugely increased in AD control and mitigated in response to GP supplementation. Although the cytokine array detects multiple types of targets, the majority of the proteins detected and analyzed in the panel were chemokines, most likely due to the fact that these are secretory proteins. Of the chemokines we were able to analyze, GROα (CXCL1), IP10 (CXCL10), MCP1 (CCL2), MIP1α (CCL3), MIP1β (CCL4) and MIP2 (CXCL2) were increased in AD serum samples and alleviated in response to GP supplementation in both prevention and intervention groups. This is an interesting finding, as collectively these cytokines contribute to inflammation and AD pathogenesis (36–40, 42). Overall, except for IL-28 (unknown role in AD) and Eotaxin [which shows conflicting result (41)], all other cytokines/chemokines results are comparable to their known previously published roles in AD pathogenesis (detailed in **Table 1**).

To identify the mechanisms of AD pathogenesis and GP supplementation responses, we implemented global proteomics analysis and identified 21 proteins that were modulated in response to GP supplementation. Though several of these are not associated directly with any known role in AD (**Table 3**), their role in other skin conditions presents an opportunity for future investigations potentially associated with AD pathogenesis (47–64). Protein network analyses using these 21 proteins, as well as other associated proteins, suggested modulation of β-catenin (CTNNB1), NRF2 (NFE2L2), VEGFA, EGFR and MMP9, which are known AD- and/or dermatological condition-associated signaling molecules. For example, β-catenin/CBP-dependent signaling has been implicated in AD pathogenesis and its inhibition prevents hapten-induced AD-like symptoms (78), activation of NRF2 signaling has been found therapeutic in AD (79), high levels of proangiogenic VEGFA are seen in AD patients (80), decreased levels of EGFR are seen in AD lesional skin (81), quercetin has been shown to inhibit MMP9 along with ERK1/2 and NF-κB pathways in AD model of human keratinocytes (82). Our proteomics analysis followed by IPA and validation identified significant inhibition in key APPs (FGA, FGB, FGG, HP, HPX and LRG1) that are linked to APR pathway. Increased HP has been seen when skin is inflamed, like that which occurs in psoriasis (83). Elevated serum level of FGA has been identified in AD patients (61). However, the role of most of the APPs are not known in AD. APR signaling is responsible for activating the system in response to stimuli such as infections, trauma, and tissue infarction, to limit the amount of tissue damage (84). APR activity is mediated by pyrogenic cytokines, such as IL-6, TNFα, and IFNγ (85). Functionally, these proteins provide pathological defense and restore homeostasis by modulating inflammation and tissue repair. Interestingly, our finding of APR involvement in AD is similar to our previous study performed in the SKH1 mouse skin cancer model, where using comparative proteomics analysis we found that GP alters a total of 14 proteins involved in the APR signaling pathway (86). Overall, this is an important finding due to the fact that though APR response aids in initial inflammatory mediation, prolonged expression of APPs can support a constitutively active inflammatory environment.

In summary, our study demonstrated that GP markedly inhibited AD-like skin lesions in both prevention and established disease settings, accompanied by decreased acanthosis and mast cell infiltration within skin, and reduction of lymphatic and/or hematopoietic expansion in spleens and lymph nodes. GP-mediated protective response was also accompanied by reduced IgE and cytokines/chemokines associated with inflammation and/or AD pathogenesis. For several of the parameters, prevention data appeared to be more promising than the intervention data, most likely due to the longer duration of GP treatment and potential inhibition of key targets during the early phases of disease progression. Proteomics analysis identified modulation in several key proteins/pathways, including APPs, in response to GP supplementation which appears to be important in AD development and progression. These observations may open up a new area of targets that have not been explored in AD management. Further studies will be required to determine how interactions of GP-modulated proteins lead to the inhibition of AD in other AD mouse models and if this applies to human patients. Overall, our study suggests that dietary grapes, containing several antioxidants in natural amalgamation, may protect against DNFB-induced AD in the prevention or established disease settings.

## Data availability statement

The original contributions presented in the study are included in the article/**Supplementary Material**, further inquiries can be directed to the corresponding author/s.

## Ethics statement

The animal study was reviewed and approved by University of Wisconsin (UW) Institutional Animal Care and Use Committee.

## Author contributions

Conceptualization: CS and NA; Data curation: CS, CM, MN, GC, SR, and RS; Formal Analysis: CS, CM, MN, GC, SR, RS, BJL, SS, and NA; Funding Acquisition and Resources: CS and NA; Investigation: CS, CM, MN, and GC; Methodology: CS, CM, MN, GC, and SR; Supervision: CS and NA; Validation: CS; Visualization: CS, CM, RS, BJL, and SS; Writing - Original Draft Preparation: CS; Writing - Review and Editing: CS, CM, MN, GC, SR, RS, BJL, SS, and NA. All authors contributed to the article and approved the submitted version.
