# Supplementary material for: Protective effects of dietary grape against atopic dermatitis-like skin lesions in NC/NgaTndCrlj mice
Source: Front Immunol. 2023 Jan 19;13:1051472. doi: 10.3389/fimmu.2022.1051472 (PMC9893861; doi:10.3389/fimmu.2022.1051472)
Supplement: Supplementary file 2 [file DataSheet_1.pdf]

Supplemental Information for:

Protective Effects of Dietary Grape Against Atopic Dermatitis-Like Skin Lesions in NC/NgaTndCrlj Mice

Chandra K. Singh[1*], Charlotte A. Mintie[1], Mary A. Ndiaye[1], Gagan Chhabra[1], Sushmita Roy[1], Ruth Sullivan[2,#], B. Jack Longley[1], Stefan M. Schieke[1], Nihal Ahmad[1,3]

Affiliation of authors:

[1] Department of Dermatology, University of Wisconsin, Madison, Wisconsin, 53705, USA

[2] Department of Comparative Biosciences, University of Wisconsin, Madison, Wisconsin, 53706, USA

[3] William S. Middleton Veterans Affairs Medical Center, Madison, Wisconsin, 53705, USA

* Correspondence:

Chandra K. Singh

E-mail: csingh@dermatology.wisc.edu

Nihal Ahmad

E-mail: nahmad@dermatology.wisc.edu

# Present address: Ruth Sullivan, Genentech, South SanFrancisco, California, USA

Short title: Dietary grapes in the management of atopic dermatitis

Supplementary Figure S1: Experimental strategies used for DNFB-induced atopic dermatitis mouse model and methodology used in the analyses.

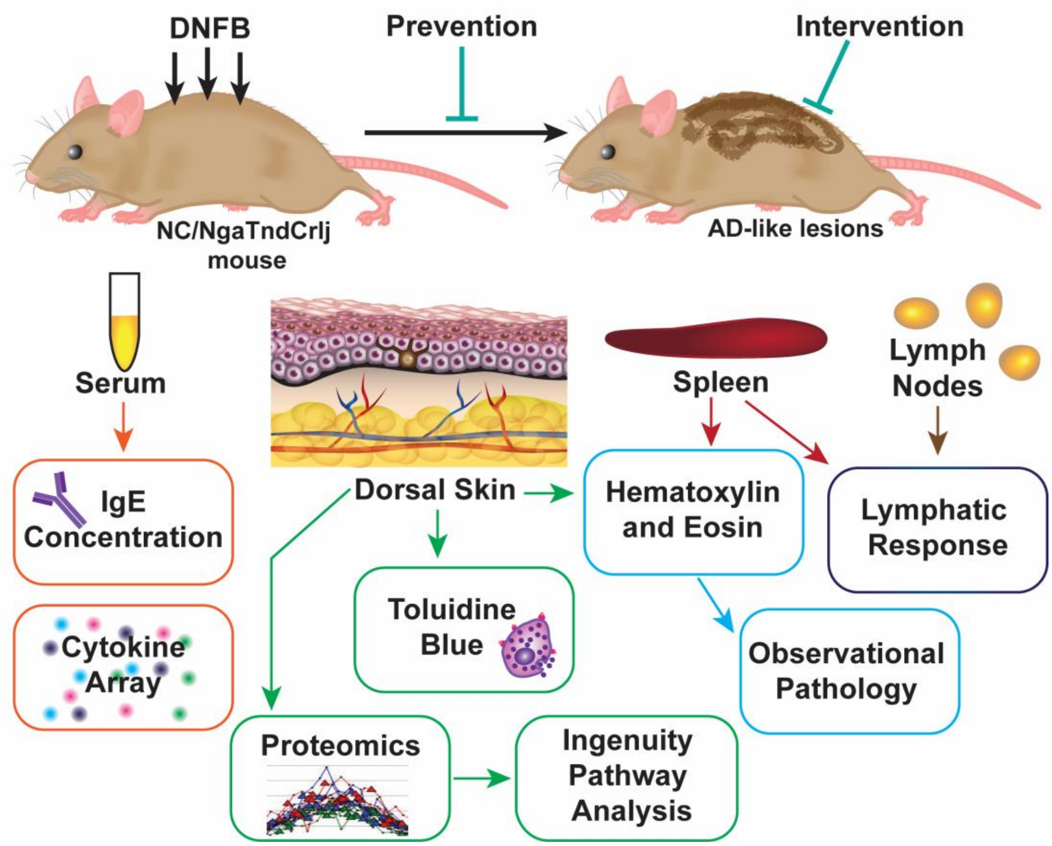

Supplementary Figure S2: Dietary grape powder (GP) inhibits atopic dermatitis-like skin lesions in NC/NgaTndCrlj mice. Images of NC/NgaTndCrlj mice from all experimental groups immediately before euthanasia. One of the mice from AD control was found dead one day before euthanasia likely due to fighting and/or excessive scratching.

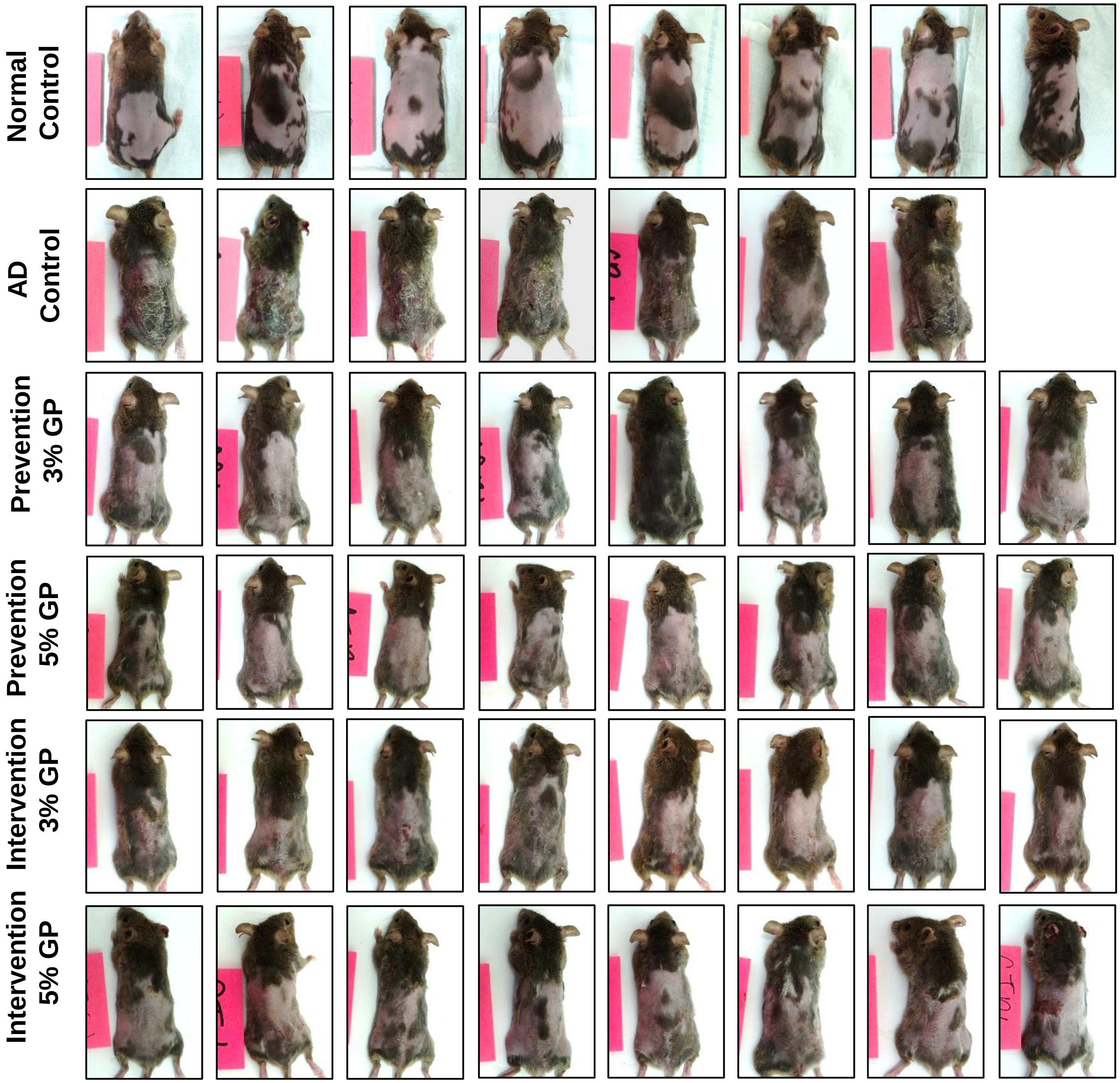

Supplementary Figure S3: No significant body weight changes were noticed in response to grape powder (GP) supplementation in NC/NgaTndCrlj mice. Weekly Body weight of NC/NgaTndCrlj mice during experimentation.

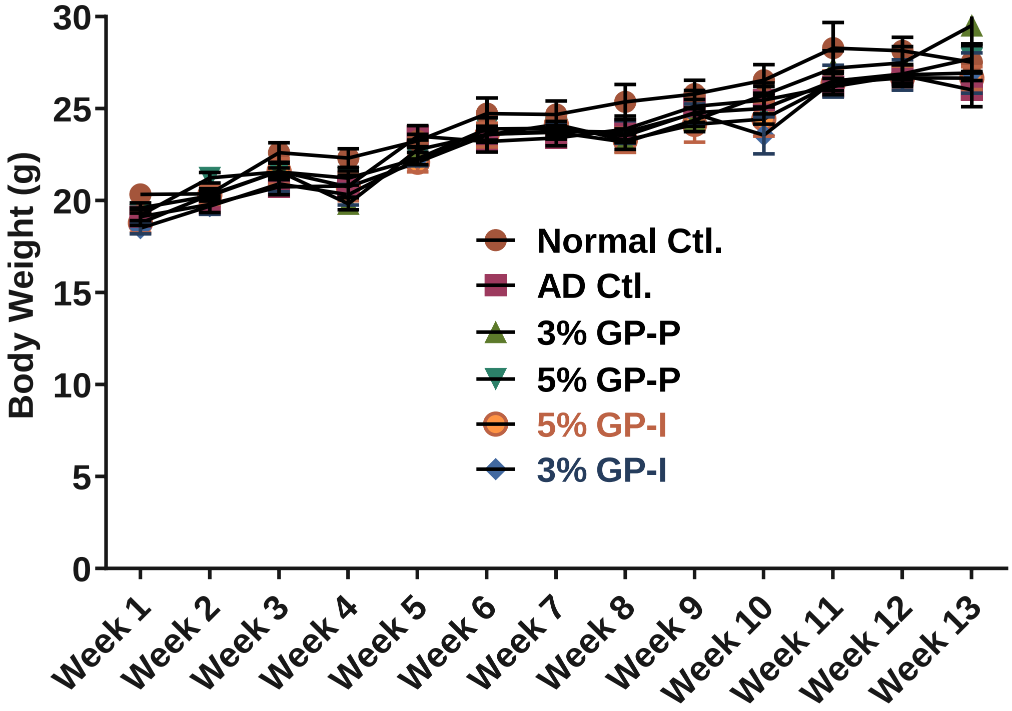

Supplementary Figure S4: Modulation in cytokines and chemokines in response to GP in serum samples of NC/NgaTndCrlj mice. The data is presented as Log2 (mean concentration of experimental groups (pg/mL)/mean concentration of AD control). NC= normal control

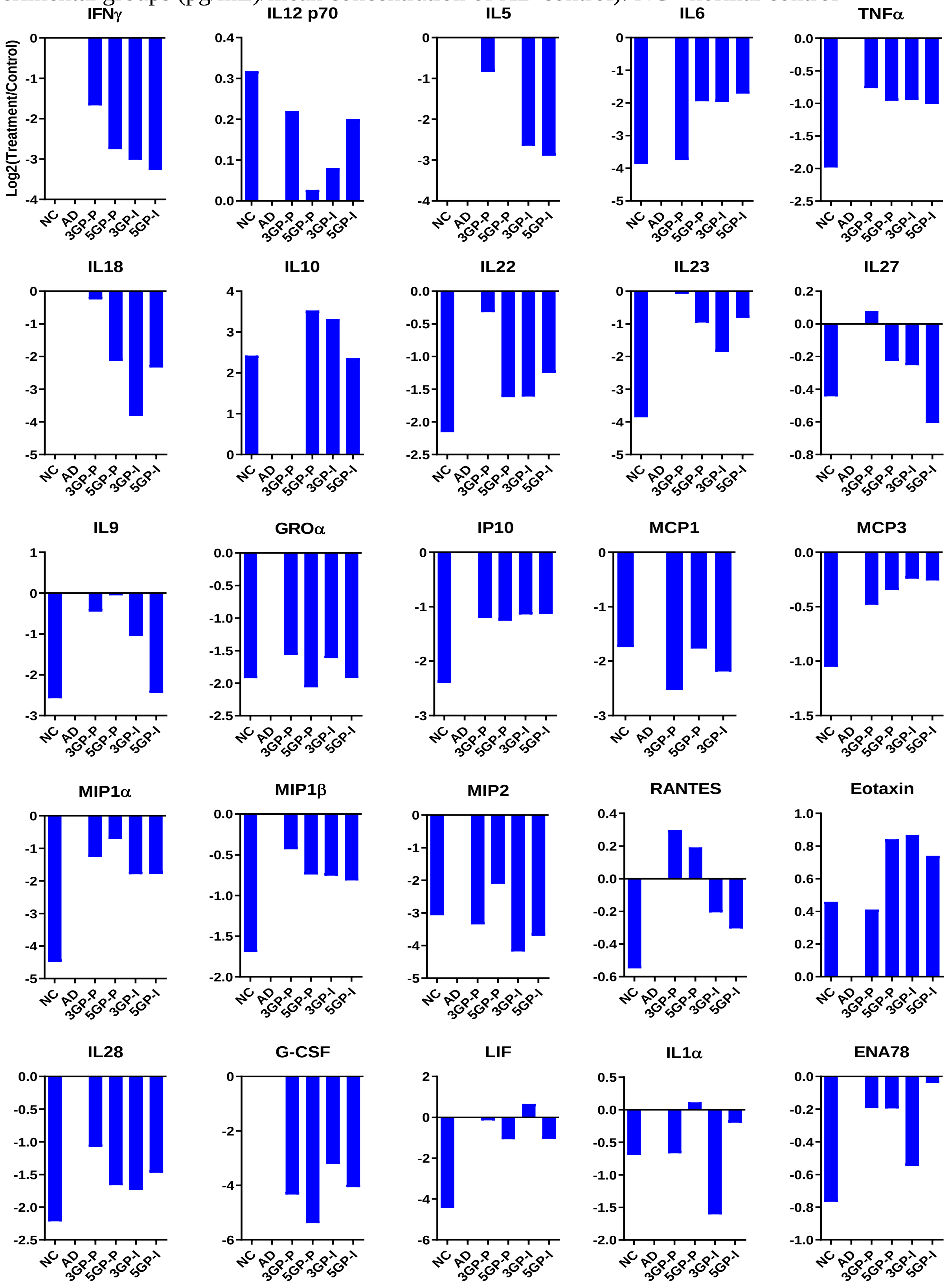

Supplementary Figure S5: Flow cytometry analysis of IL4 expression in lymph nodes. IL4 was measured in lymph nodes using Flow cytometry as described in the 'Materials and Methods' section. The data were analyzed using one-way ANOVA with Dunnett's multiple comparison test and presented as mean ± SEM with statistical significance compared to AD control (*p<0.05, **p<0.01).

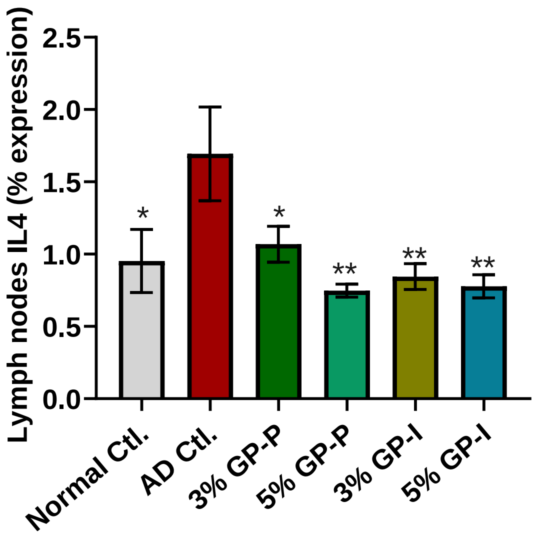

Supplementary Figure S6: RT-qPCR analysis of TSLP. ACTB was used as an endogenous control. The data were analyzed using one-way ANOVA with Dunnett's multiple comparison test and presented as mean ± SEM with statistical significance compared to AD control (*p<0.05, ****p<0.0001).

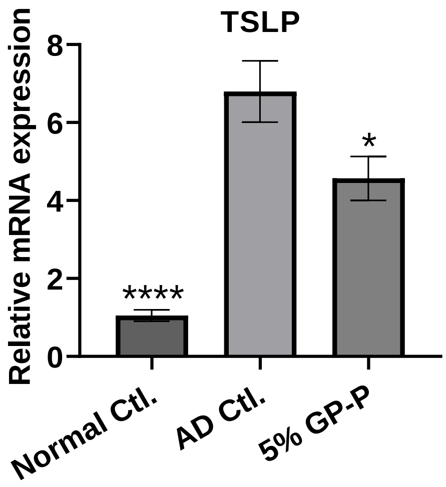

Supplementary Figure S7: Quantitative proteomics analysis identified key atopic dermatitis-associated signaling in response to grape powder (GP) supplementation. (A) Summary of quantitative proteomics approach in protocol and analysis. **(B)** Base peak chromatograms after Sieve alignment. **(C)** Sieve alignment score of analyzed samples.

A

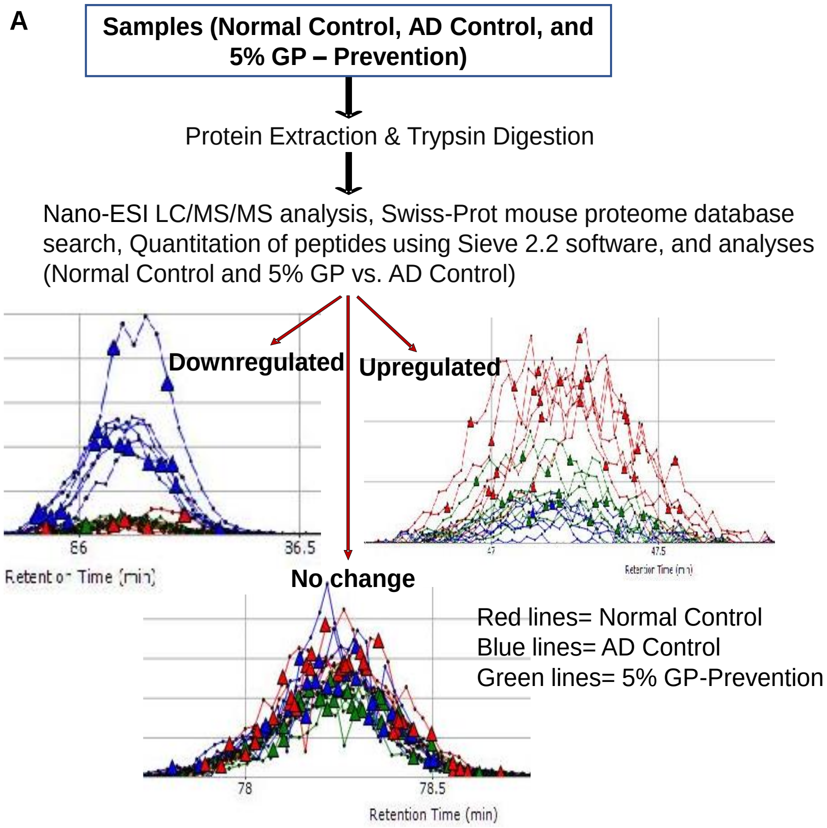

B Samples and Sieve Alignment Score

Samples	Sieve Score
normal control 1	0.773
normal control 2	0.776
normal control 3	0.731
normal control 4	0.794
normal control 5	0.766
normal control 6	0.801
AD control 1	0.799
AD control 2	1.0
AD control 3	0.859
AD control 4	0.879
AD control 5	0.879
AD control 6	0.867
5GP 1	0.807
5GP 2	0.75
5GP 3	0.803
5GP 4	0.771
5GP 5	0.763
5GP 6	0.827

C Base peak chromatograms after alignment (SIEVE 2.2, 18-120 min retention time)

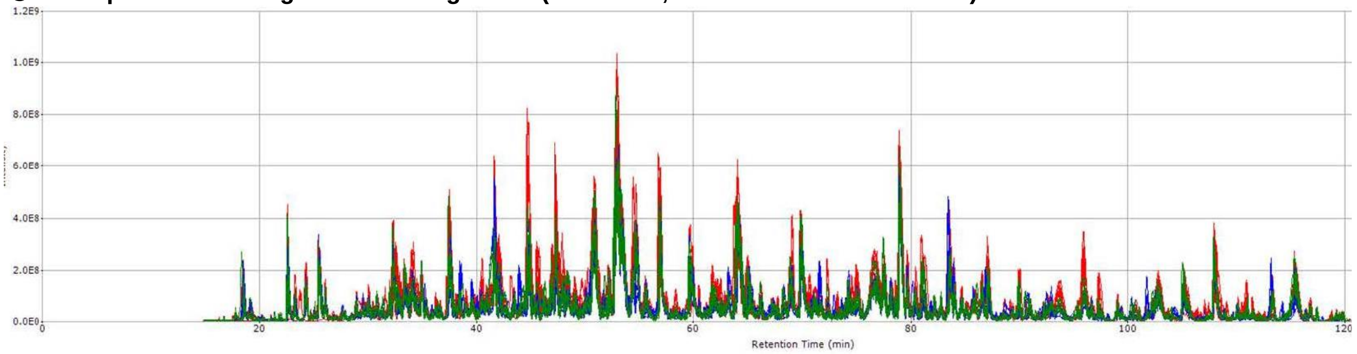

Table S1: Details about the antibodies and lysate concentrations used for the quantitative immuno-detection analyses using Jess ProteinSimple in a 12-230 kDa microplate.

Antibody	Molecular weight (KDa)	Supplier	Catalog number	Antibody dilution	Lysate concentration
FGA	60	Proteintech	20645-1-AP	1:50	1.0 ug/uL
FGB	50	Proteintech	16747-1-AP	1:200	0.5ug/uL
FGG	50	Invitrogen	PA5-21968	1:50	1.0 ug/uL
HP	42	Invitrogen	PA5-79391	1:25	1.0 ug/uL
HPX	70	Invitrogen	PA5-96367	1:50	1.0 ug/uL

Table S2: Primer sequences used for RT-qPCR analyses

Gene	Amplicon size (bp)	Primer orientation	Primer Sequence (5' - > 3')	Length	Tm	PrimerBank ID
ACTB	154	F	GGCTGTATTCCCCTCCATCG	20	61.8	6671509a1
		R	CCAGTTGGTAACAATGCCATGT	22	61.1	
LRG1	190	F	TTGGCAGCATCAAGGAAGC	19	60.7	16418335a1
		R	CAGATGGACAGTGTCGGCA	19	61.7	
TSLP	128	F	GCTAAGTTCGAGCAAATCGAGG	22	61.3	283945612c2
		R	GCCAGGGATAGGATTGAGAGTA	22	60.2	
IL6	131	F	CTGCAAGAGACTTCCATCCAG	21	60.1	
		R	AGTGGTATAGACAGGTCTGTTGG	23	60.8	13624310c1
